# Plasma Epstein-Barr Virus MicroRNA BART8-3p as a Diagnostic and Prognostic Biomarker in Nasopharyngeal Carcinoma

**DOI:** 10.1093/oncolo/oyac024

**Published:** 2022-03-05

**Authors:** Cheng Lin, Keyu Lin, Bin Zhang, Ying Su, Qiaojuan Guo, Tianzhu Lu, Yuanji Xu, Shaojun Lin, Jingfeng Zong, Jianji Pan

**Affiliations:** 1 Department of Radiation Oncology, Fujian Medical University Cancer Hospital & Fujian Cancer Hospital, Fuzhou, People’s Republic of China; 2 The School of Clinical Medicine and Fujian Medical University, Fuzhou, People’s Republic of China; 3 Department of Radiation Biology, Fujian Medical University Cancer Hospital & Fujian Cancer Hospital, Fuzhou, People’s Republic of China; 4 Department of Genetics and Genomic Sciences, Icahn Institute of Genomics and Multiscale Biology, Mount Sinai Center for Transformative Disease Modeling, Icahn School of Medicine at Mount Sinai, New York, NY, USA

**Keywords:** nasopharyngeal carcinoma, Epstein-Barr virus, BART8-3p, biomarker, metastasis

## Abstract

**Background:**

Nasopharyngeal carcinoma is an Epstein-Barr virus (EBV)-associated tumor that is highly common in southern China. Our previous sequencing data demonstrated that the EBV-encoded microRNA BART8-3p was most upregulated in nasopharyngeal carcinoma (NPC) and was closely associated with the metastasis of NPC. However, the values of plasma BART8-3p in NPC patients have not yet been well characterized.

**Material and Methods:**

We quantified plasma BART8-3p expression by quantitative real-time PCR in 205 newly diagnosed NPC patients. Kaplan-Meier analysis was used to compare overall survival (OS), distant metastasis-free survival (DMFS), and locoregional relapse-free survival (LRRFS) between the groups.

**Results:**

Plasma pretreatment BART8-3p was highly expressed in NPC patients compared with healthy controls. Pretreatment BART8-3p yielded a 92% predictive value for detecting NPC. Importantly, BART8-3p decreased dramatically after therapy relative to pretreatment levels. High levels of pretreatment or post-treatment BART8-3p were associated with worse OS, DMFS, and LRRFS. Multivariate analysis showed that high pretreatment or post-treatment BART8-3p was an independent unfavorable prognostic marker for OS (HR 3.82, 95% CI 1.77-8.24, *P* = .001 or HR 2.74, 95% CI 1.27-5.91, *P* = .010), DMFS (HR 2.82, 95% CI 1.36-5.85, *P* = .005 or HR 3.27, 95% CI 1.57-6.81, *P* = .002), and LRRFS (HR 1.94, 95% CI 1.12-3.35, *P* = .018 or HR 2.03, 95% CI 1.14-3.62, *P* = .016) in NPC. Subgroup analysis revealed that for patients with locally advanced NPC with high levels of pretreatment BART8-3p (*n* = 58), more cycles of chemotherapy (≥6 cycles) tended to prolong OS (*P* = .070). Over 50% (6/11) patients with high levels of post-treatment BART8-3p presented distant metastasis.

**Conclusion:**

Plasma BART8-3p is a promising biomarker for the detection and prognosis of NPC.

**Implications for Practice**

Nasopharyngeal carcinoma is an Epstein-Barr virus-associated tumor. Majority of patients are initially diagnosed with locally advanced nasopharyngeal carcinoma, resulting in a relatively poor prognosis. Our study found that plasma pretreatment BART8-3p can well-distinguish NPC patients from healthy controls. BART8-3p decreased dramatically after therapy. More importantly, plasma pretreatment and post-treatment BART8-3p are independent prognostic biomarker for predicting survival and the risk of recurrence and metastasis. Application of plasma BART8-3p in early recognition of disease onset and monitoring of therapy warrant further exploration and evaluation.

## Introduction

Nasopharyngeal carcinoma (NPC) is responsible for a significant proportion of cancer-related deaths in southern China, and the incidence rate can be up to 30 per 100 000 person-years.^1^ NPC is highly sensitive to radiotherapy (RT), and patients with early NPC can have a 5-year survival rate of over 90% by intensity-modulated radiotherapy (IMRT).^2^ However, more than 70% of patients are initially diagnosed with locally advanced NPC, leading to a relatively poor prognosis.^3,4^ Thus, there is a pressing need to explore biomarkers to guide early screening and aid clinical decision making.

Epstein-Barr virus (EBV) infection is an extremely significant step in the pathogenesis of NPC and can be observed in nearly 100% of nonkeratinizing NPCs in endemic areas.^1^ Better yet, EBV has rarely been examined in normal nasopharyngeal epithelium.^5^ Thus, detecting EBV-associated products is a feasible and promising way to identify biomarkers that could assist in the diagnosis of NPC. Epstein-Barr virus antibodies, especially EBV capsid antigen (VCA)/IgA, were first identified to be a potential prognostic indicator for therapeutic response evaluations and early screening in NPC.^6,7^ However, the sensitivity and specificity were relatively low for VCA/IgA, and the accumulated probability of developing NPC in subjects with positive VCA/IgA antibodies was only 7.16%.^8^ Currently, EBV DNA is regarded as a significant biomarker for the early detection, prognostication, and monitoring of therapeutic response in clinical practice.^9-11^ However, a standardization of EBV DNA quantification is lacking, and the level of EBV DNA can be affected by a multiplicity of factors, limiting its application.^12-14^ In addition, the mechanisms of how EBV DNA are generated and released into the bloodstream remain unclear. Thus, novel biomarkers for NPC are of great interest and warrant further study.

MicroRNAs (miRNAs) are small, noncoding RNAs (~22 nucleotides) that bind to the 3ʹ-untranslated regions of mRNA, leading to the translational degradation or repression of mRNA.^15^ Epstein-Barr virus miRNAs were first identified in 2004, and later studies confirmed that EBV encodes 2 transcripts, including BamHI-A region rightward transcripts (BARTs) and BamHI fragment H rightward open reading frame 1 (BHRF1).^16^ Epstein-Barr virus BARTs encode 44 mature miRNAs (BART miRNAs), and accumulating evidence suggests that BART miRNAs are abundantly expressed in EBV-positive C666-1 cells, NPC plasma samples and issues,^17-22^ suggesting that BARTs play an important role in the development of NPC. Our previous study showed that compared with healthy controls (HCs), BART8-3p was the most highly overexpressed BART miRNA in NPC tissues by microRNA sequencing, and BART8-3p promoted metastasis by activating the NF-κB and Erk1/2 pathways in vitro.^23^ Unfortunately, the expression and clinical significance of plasma circulating BART8-3p have not been further explored and are not well established. Therefore, in this study, we aimed to evaluate the potential value of BART8-3p as a novel biomarker in patients with NPC.

## Materials and Methods

### Patients and Plasma Collection

A total of 205 newly diagnosed NPC patients without distant metastasis were enrolled between January 2013 and April 2014, and all patients received IMRT. Patients with NPC were reclassified by the new AJCC/UICC 8th Edition Staging System. Blood samples from 96 healthy volunteer donors were collected from our physical examination center for control. Whole blood samples were collected in EDTA-containing tubes, and plasma was separated by centrifugation at 3000*g* for 10 minutes and then stored at −80 °C for further use. Blood samples before receiving any antitumor treatment and within 3 days after completing RT were collected and defined as pretreatment and post-treatment, respectively. This study was performed in accordance with the Declaration of Helsinki and was approved by the Ethical Review Committee of Fujian Cancer Hospital (approval no. 2015-010-02). Our study was carried out according to the Reporting Recommendations for Tumor Marker Prognostic Studies.^24^

### Treatment and Follow-Up

All NPC patients without distant metastasis were treated with definitive IMRT. The IMRT protocol was described in detail in our previously published article.^2^ Stage I NPC is treated with RT alone, stage II NPC is treated with concurrent chemoradiotherapy, and stages III-IVA NPC is treated with platinum-based chemotherapy and RT. The platinum-based chemotherapy regimen typically consists of gemcitabine or paclitaxel plus cisplatin or nedaplatin, and the concurrent chemotherapy regimen includes cisplatin or nedaplatin. The median follow-up time was 69.0 months. The median follow-up time was 69.0 months. The overall survival (OS) was calculated from the day of diagnosis to the day of death from any cause. Distant metastasis-free survival (DMFS) was calculated from the day of diagnosis to the time of distant metastasis. Locoregional relapse-free survival (LRRFS) was calculated from the day of diagnosis to the time of locoregional failure. The follow-up schedule was also described in a previously published study.^25^

### RNA Extraction, cDNA Synthesis, and miRNA Quantitative Analysis

Isolation and purification of total plasma were conducted using the RNA miRNeasy Serum/Plasma Advanced Kit (Qigen, Germany), and steps were performed entirely according to the protocol. Briefly, specialized buffer was added in succession, and then the mixture was centrifuged to remove protein precipitate. Then, isopropanol was added to the supernatant to bind total RNA. Finally, the mixture was washed and eluted with RNase-free water. Final products were reserved in -20 °C refrigerator. Reverse transcription of miRNA was conducted using the TaqMan MicroRNA Reverse Transcription Kit (Applied Biosystems, Thermo Fisher Scientific, USA) with the following conditions: 16 °C for 30 minutes, 42 °C for 30 minutes, 85 °C for 5 minutes. Quantitative real-time PCR was carried out using TaqMan Universal Master Mix II, no UNG (Applied Biosystems, Thermo Fisher Scientific, USA) on 7200 real-time PCR systems (Applied Biosystems, Thermo Fisher Scientific, USA) with the following conditions: 95 °C for 10 minutes, 45 cycles of 15 s at 95 °C and 1 minutes at 60 °C. A standard curve was established by serially diluted miRNA mimics. Ce_miR-39 was introduced to monitor miRNA purification and amplification, and data from PCR amplification were normalized by Ce-miR-39 amplification. The specific information of TaqMan probes, primers for reverse transcription, and PCR are attached in Supplementary Table S1.

### Statistics

All statistical analyses were performed using SPSS version 24.0 and GraphPad Prism 8. Associations between BART miRNA levels and clinical characteristics were examined using Spearman’s relative test. The paired plasma samples obtained before and after treatment were compared using the Wilcoxon test. The cutoff value of plasma miR-BART8-3p in NPC versus HCs was derived by receiver operating characteristic (ROC) curves with Youden’s index. The median expression of BART8-3p is used as the cutoff value between high level and low levels. Survival was examined by the Kaplan-Meier (K-M) method, and differences were calculated by the log-rank test. Multivariable analyses were conducted with a Cox proportional hazards model. All *P* values were 2 sided.

## Results

### Higher Circulating BART8-3p Levels Can Distinguish NPC Patients from Healthy Controls

Epstein-Barr virus BART miRNA expression was profiled in NPC and normal nasopharyngeal mucosal tissues in our previous study.^23^ Our results suggested that BART miRNA levels were significantly higher in NPC patients than in HCs. In particular, BART8-3p was the most upregulated EBV BART miRNA, indicating that BART8-3p might be a potential biomarker in NPC.

**Figure 1.**
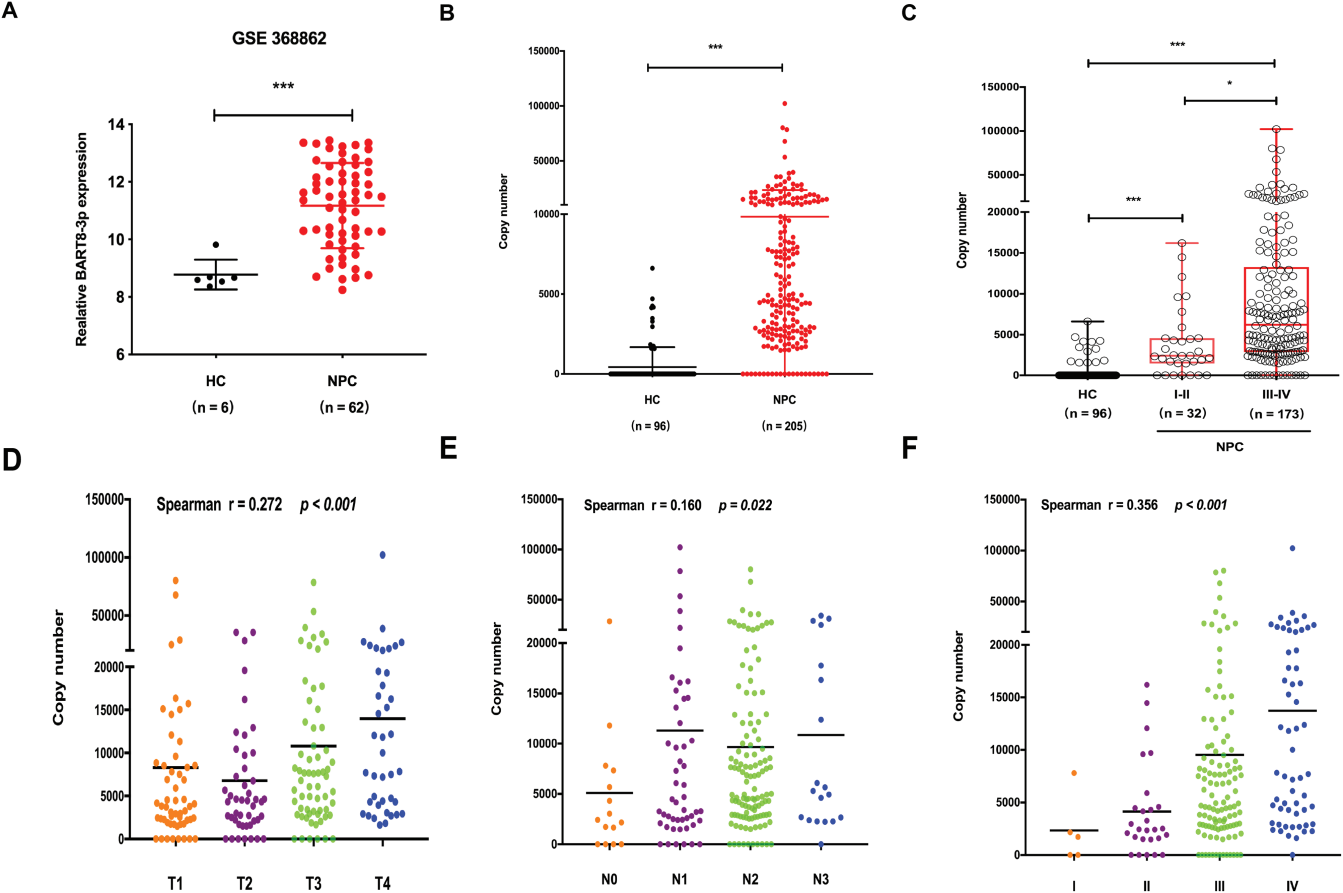
Plasma BART8-3p in healthy controls (HCs) and NPC patients. (**A**) BART8-3p expression in NPC tissues in another microRNA microarray (GSE36682). (**B**, **C**) Levels of plasma BART8-3p in HCs and NPC patients. (**D**, **E**, **F**) Levels of BART8-3p in different NPC tumor stages, node stages and TNM stages. ∗ *P < .*05, ∗∗ *P < .*01 and ∗∗∗ *P < .*001.

Another microRNA microarray (GSE 368862) from a different cancer center was used to confirm our previous findings in NPC tissues, and the results also showed that BART8-3p was overexpressed in NPC tissues compared with HCs (Fig. 1A). Then, we further evaluated BART8-3p expression in NPC patient plasma. The clinicopathological features of 205 NPC patients stratified by pretreatment or post-treatment BART8-3p levels are shown in Table 1, with a median age of 48.7 years (from 21 to 79 years). The data revealed that pretreatment BART8-3p levels were highly elevated in 61 (29.76%) NPC patients (Table 1) and that BART8-3p was more abundant in the circulation in NPC patients than in HCs (*P < .*001) (Fig. 1B). Further analysis indicated that late-stage NPC (n =173) had remarkably higher levels of BART8-3p than early-stage NPC patients (*n* = 32) and HCs (*n* = 96; Fig. 1C). Consistent with the chi-square test in Table 1, we found that higher concentrations of pretreatment BART8-3p were closely associated with advanced T stage and TNM stage (Fig. 1D-F), suggesting that BART8-3p is strongly related to NPC progression. In addition, high levels of post-treatment BART8-3p were found only in locally advanced NPC (LA-NPC) patients.

**Table 1.** Characteristics of 205 patients with nasopharyngeal carcinoma grouped by plasma BART8-3p expression level.

Variables	Overall	Pretreatment BART8-3p		*P*	Post-treatment BART8-3p		*P*
		Low (*n* = 144)	High (*n* = 61)		Low (*n* = 194)	High (*n* = 11)	
Sex				.600			.819
Male	146	101	45		139	7	
Female	59	43	16		55	4	
Age at diagnosis				.596			.952
≤50 years	110	79	31		104	6	
>50 years	95	65	30		90	5	
Histology				.834			.802
KSCC	2	1	1		2	0	
NKDC	27	19	8		25	2	
NKUC	176	124	52		167	9	
T classification				.006			.327
T1-2	101	80	21		94	7	
T3-4	104	64	40		100	4	
N classification							.511
N0-1	65	47	18	.660	63	2	
N2-3	140	97	43		131	9	
AJCC stage				.006			.299
I-II	32	29	3		32	0	
III-IV	173	115	58		162	11	
Chemotherapy^a^				.293			.760
<4 cycles	75	56	19		70	5	
≥4 cycles	130	88	42		124	6	

Chemotherapy: including induction chemotherapy, concurrent chemoradiotherapy, and adjuvant chemotherapy.

KSCC, keratinizing squamous cell carcinoma; NKDC, nonkeratinizing differentiated carcinoma; NKUC, nonkeratinizing undifferentiated carcinoma; AJCC, American Joint Committee Cancer.

**Figure 2.**
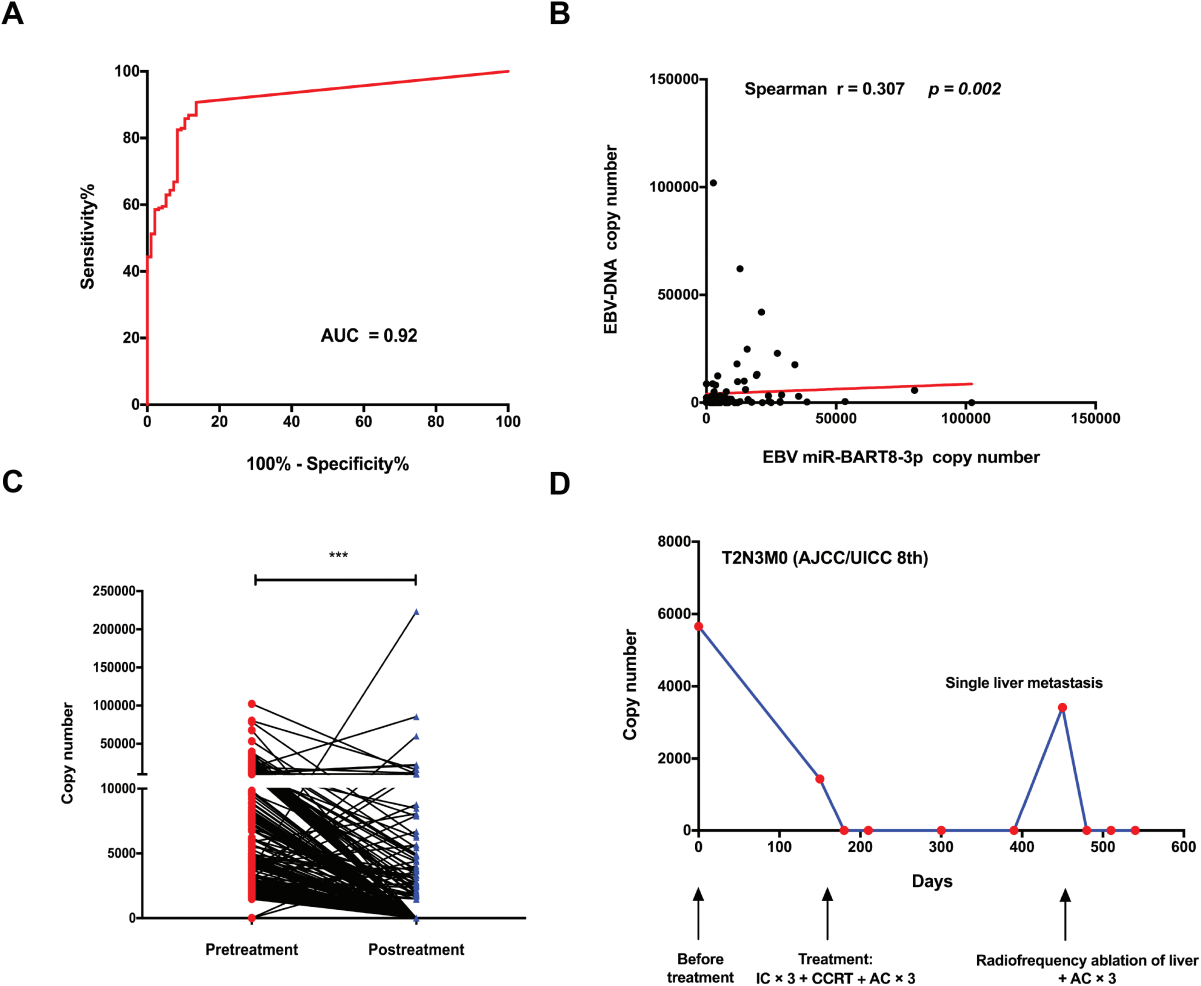
Clinical application of plasma BART8-3p in NPC. (**A**) ROC curve analysis of BART8-3p for discriminating NPC from healthy controls. (**B**) Correlation of BART8-3p and EBV DNA load among patients with NPC. (**C**) Change in BART8-3p in NPC patients after therapy. (**D**) Evolution of BART8-3p in a locally advanced NPC patient who developed liver metastasis 10 months after completing the entire therapy. ∗∗∗ *P < .*001.

To further assess the diagnostic performance of BART8-3p, we used a ROC curve and found that BART8-3p had a predictive value of 92% (95% CI: 0.88-0.95; Fig. 2A). Using a cutoff value of 1668 copies/mL, the sensitivity and specificity for identifying a patient with NPC were 87.8% and 89.6%, respectively. Interestingly, a statistically significant positive correlation was observed between circulating BART8-3p and plasma EBV DNA load (Spearman *r* = .307, *P = .*002; Fig. 2B).

Taken together, these results suggest that circulating miR-BART8-3p originating from NPC will enter the circulation and could potentially serve as a molecular marker for the detection of NPC.

### Circulating BART8-3p in NPC Patients Before and After Treatment

Even with the advancement of IMRT, approximately 5%-15% of patients will suffer from local recurrence, and 15%-30% will develop distant metastasis.^26,27^ Thus, identifying noninvasive biomarkers to monitor and predict the progression of NPC is of utmost importance. Emerging studies have shown microRNA levels in plasma to be a useful marker for predicting therapeutic benefits.^28,29^ We therefore determined whether BART8-3p could be used as an alternative to EBV DNA to predict therapeutic effects on NPC.^9^

To confirm our speculation, we further compared the levels of BART8-3p before any antitumor treatment (pretreatment) and after complete therapy (post-treatment) in a subset of 205 NPC patients. It was interesting to note that miR-BART8-3p was completely undetectable or greatly ­decreased in the majority of patients after RT (*P < .*001) (Fig. 2C). A few patients had reduced levels of BART8-3p or even presented with increased levels of BART8-3p. Examination of the clinical data indicated that among the 11 patients with high post-treatment BART8-3p levels, 7 experienced local failure or distant metastasis.

A representative case of classic NPC is described to better illustrate the dynamic change in BART8-3p (Fig. 2D). A 48-year-old man presented with a T2N3M0 EBV-positive nonkeratinizing undifferentiated NPC. He received 3 cycles of induction chemotherapy followed by concurrent chemoradiotherapy and 3 cycles of adjuvant chemotherapy. The concentration of miR-BART8-3p was 5656.67 copies/mL before treatment, and it was reduced to 1436.98 copies/mL after the completion of treatment and then decreased to 0 copies/mL after 1 month at the first follow-up. After approximately 10 months, this patient developed liver metastasis at a single site, and surprisingly, the concentration of BART8-3p had risen robustly to 3415.00 copies/mL. Then, the patient received radiofrequency ablation^30^ followed by 3 cycles of adjuvant chemotherapy (cisplatin/paclitaxel). Interestingly, the concentration of BART8-3p quickly decreased back to 0 copies/mL after RFA, and the patient was in great condition at the last follow-up.

Taken together, our findings indicate that circulating BART8-3p could serve as a valuable molecular biomarker for monitoring therapeutic efficacy.

### Correlation of Circulating miR-BART8-3p and Prognosis in 205 NPC Patients

**Figure 3.**
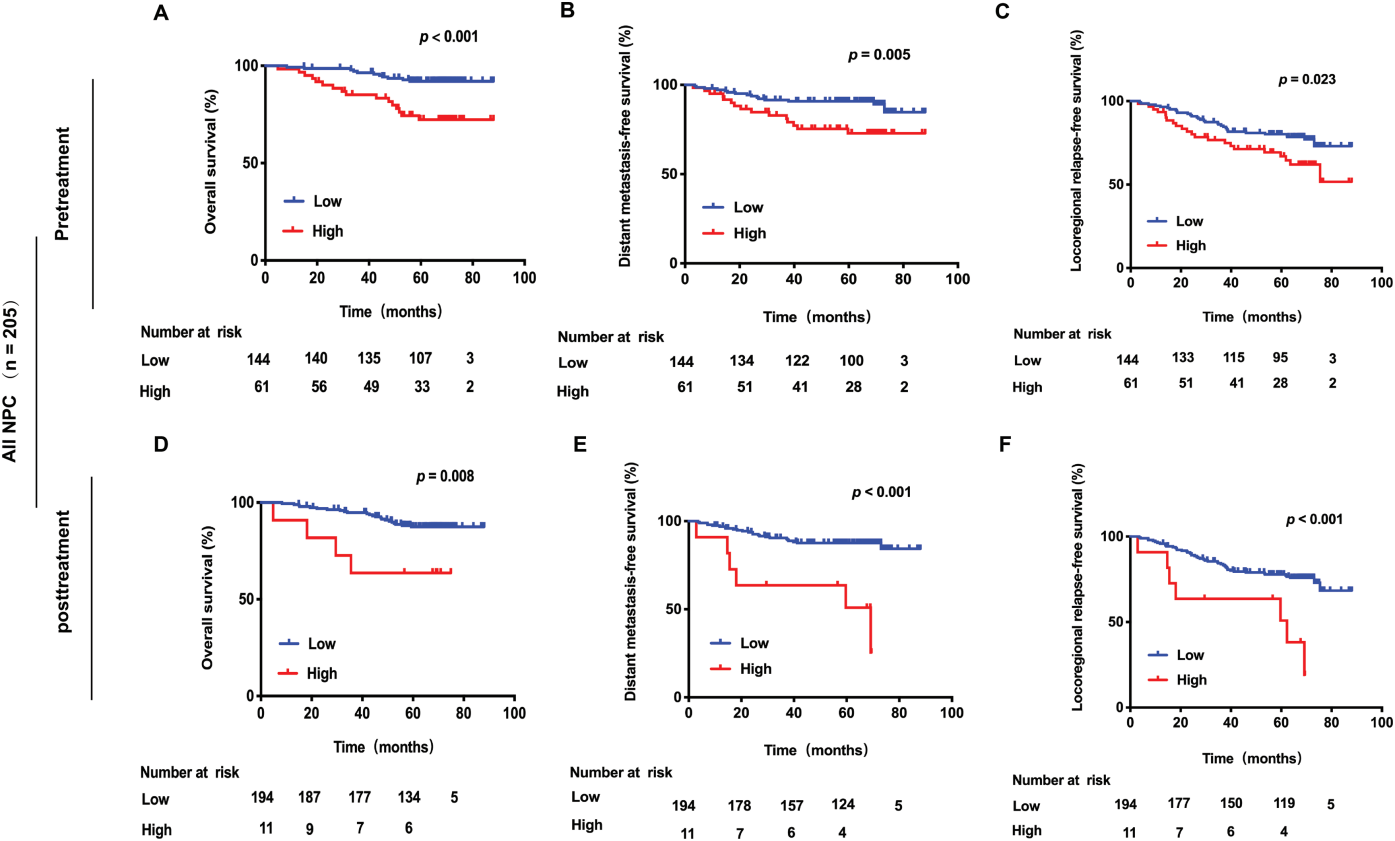
Kaplan-Meier estimates of overall survival (OS), distant metastasis-free survival (DMFS), and locoregional relapse-free survival (LRRFS) according to pretreatment or post-treatment BART8-3p in 205 NPC patents. Comparison of OS (**A**), DMFS (**B**), and LRRFS (**C**) according to BART8-3p pretreatment (upper row). Comparison of OS (**D**), DMFS (**E**), and LRRFS (**F**) according to BART8-3p post-treatment (lower row).

To address whether BART8-3p levels can be used as a predictor of patient outcome, we performed K-M analysis. The median expression of BART8-3p was used as the cutoff value between high and low levels. First, we explored the values of pretreatment BART8-3p. Compared to patients with low pretreatment BART8-3p levels, patients with high pretreatment BART8-3p levels had significantly worse OS, DMFS, and LRRFS; Fig. 3A-C). In addition, multivariate analysis showed that low levels of pretreatment BART8-3p were closely associated with significantly longer OS (HR 3.82, 95% CI 1.77-8.24; *P* = .001), DMFS (HR 2.82, 95% CI 1.36-5.85; *P* = .005) and LRRFS (HR 1.94, 95% CI 1.12-3.35; *P* = .018) (Table 2).

**Table 2.** Multivariate analysis of overall survival (OS), distant metastasis-free survival (DMFS), and locoregional relapse-free survival (LRRFS) in 205 patients with nasopharyngeal carcinoma according to the levels of BART8-3p.

Variables	Pretreatment BART8-3p						Post-treatment BART8-3p					
	OS	*P*	DMFS	*P*	LRRFS	*P*	OS	*P*	DMFS	*P*	LRRFS	*P*
	HR(95% CI)		HR(95% CI)		HR(95% CI)		HR(95% CI)		HR(95% CI)		HR(95% CI)	
Sex		.021		.012		.043		0.021		.013		.050
Male vs Female	0.18 (0.04-0.77)		0.08 (0.01-0.58)		0.49 (025-0.99)		0.18 (0.04-0.77)		0.08 (0.01-0.59)		0.50 (0.25-0.99)	
BART8-3p		.001		.005		.018		.010		.002		.016
Low VS high	3.82 (1.77-8.24)		2.82 (1.36-5.85)		1.94 (1.12-3.35)		2.74 (1.27-5.91)		3.27 (1.57-6.81)		2.03 (1.14-3.62)	

The clinical significance of post-treatment BART8-3p was also investigated. K-M analysis found that high concentrations of BART8-3p were related to poorer OS, DMFS and LRRFS (Fig. 3D-F). Most importantly, the multivariate analysis results confirmed that high post-treatment BART8-3p levels were an independent unfavorable predictor for OS (HR 2.74, 95% CI 1.27-5.91; *P* = .010), DMFS (HR 3.27 95% CI 1.57-56.81; *P* = .002) and LRRFS (HR 12.03, 95% CI 1.14-3.62; *P* = .016) (Table 2).

Collectively, our data indicate that high expression of BART8-3p is linked to inferior survival and poor prognosis in NPC patients.

### Evaluation of Circulating miR-BART8-3p in 173 Locally Advanced NPC Patients

The majority of patients (≥70%) presented with locally advanced NPC, leading to a high rate of locoregional relapse and poorer survival. We therefore wondered whether the levels of BART8-3p could be a biomarker in guiding the clinical practice of locally advanced NPC (LA-NPC). To further validate this hypothesis, 173 patients with LA-NPC (84.4%) among the 205 NPC patients were included and analyzed.

**Figure 4.**
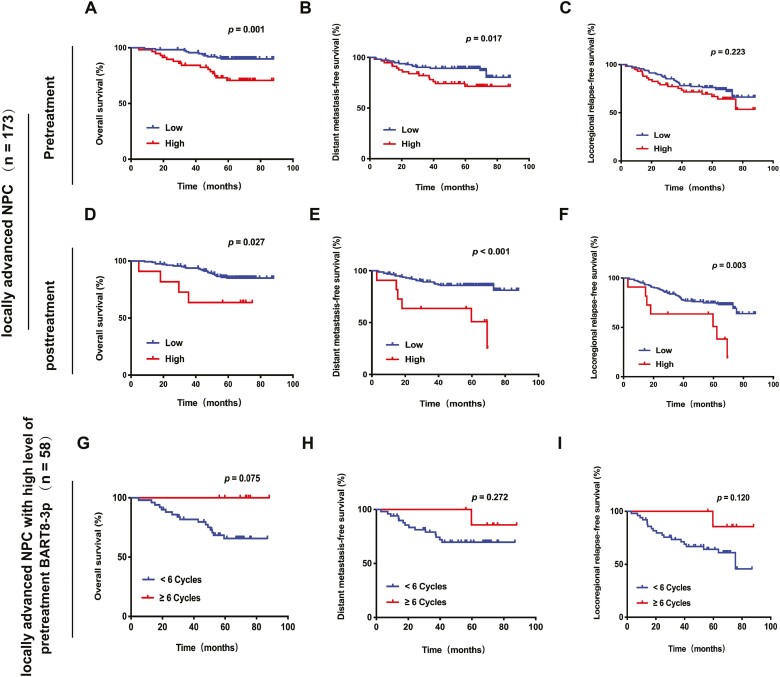
Kaplan-Meier estimates of overall survival (OS), distant metastasis-free survival (DMFS), and locoregional relapse-free survival (LRRFS) according to pretreatment or post-treatment BART8-3p in 173 locally advanced NPC patents from 205 NPC patients. Comparison of OS (**A**), DMFS (**B**), and LRRFS (**C**) according to pretreatment with BART8-3p (top row). (**D**, **E**, **F**) Comparison of OS, DMFS and LRRFS according to post-treatment BART8-3p (middle row). Comparison of OS (**G**), DMFS (**H**), and LRRFS (**I**) in 58 locally advanced NPC patients with high levels of pretreatment BART8-3p (bottom row).

Regarding the levels of pretreatment BART8-3p, LA-NPC with high levels of pretreatment BART8-3p had shorter OS and DMFS, while no statistically significant difference was observed with respect to LRRFS (Fig. 4A-C). Multivariable analysis demonstrated that high levels of pretreatment BART8-3p served as an independent unfavorable prognostic marker for OS (HR 3.12, 95% CI 1.45-6.74; *P* = .004) and DMFS (HR 1.26, 95% CI 0.110-0.601; *P < .*001) but not LRRFS (Table 3). However, LA-NPC with high levels of post-treatment BART8-3p had shorter OS, DMFS and LRRFS times (Fig. 4D-F). The multivariable analysis confirmed that high levels of post-treatment BART8-3p were associated with worse DMFS (HR 2.68, 95% CI 1.27-5.65; *P* = .009) but not OS (HR 2.13, 95% CI 0.99-4.60; *P* = .054) or LRRFS (HR 1.75, 95% CI 0.98-3.16; *P* = .061). Among 173 patients, nearly one-third (58/173) had a high concentration of pretreatment BART8-3p. Subgroup analysis demonstrated that for patients with a high concentration of pretreatment BART8-3p, receiving more than 6 cycles of chemotherapy, including induction chemotherapy, concurrent systemic therapy and adjuvant chemotherapy, tended to prolong OS (*P* = .075) (Fig. 4G-I). Of the 173 LA-NPC patients, only 6.36% (11/173) had high post-treatment levels of BART8-3p, of which 50% (6/11) had distant metastasis.

**Table 3.** Multivariate analysis of overall survival (OS), distant metastasis-free survival (DMFS, and locoregional relapse-free survival (LRRFS) in 173 patients with locally advanced nasopharyngeal carcinoma according to the levels of BART8-3p.

Variables	Pretreatment BART8-3p						Post-treatment BART8-3p					
	OS	*P*	DMFS	*P*	LRRFS	*P*	OS	*P*	DMFS	*P*	LRRFS	*P*
	HR(95% CI)		HR(95% CI)		HR(95% CI)		HR(95% CI)		HR(95% CI)		HR(95% CI)	
Sex		.018		.012		.047		.018		.014		.057
Male vs female	0.18 (0.04-0.74)		0.08 (0.01-0.57)		0.49 (0.25-0.99)		0.17 (0.04-0.74)		0.08 (0.01-0.60)		0.51 (0.25-1.02)	
BART8-3p		.004		.020		.197		.054		.009		.061
Low vs high	3.12 (1.45-6.74)		2.41 (1.15-5.07)		1.45 (0.82-2.56)		2.13 (0.99-4.60)		2.68 (1.27-5.65)		1.75 (0.98-3.16)	

Collectively, the results of our study underscore the importance of BART8-3p as a potential biomarker for patients with LA-NPC, especially in the survival of OS and DMFS.

## Discussion

As NPC is relatively asymptomatic, the majority of NPC patients present with locally advanced disease or develop distant metastasis at diagnosis. Therefore, the identification of simple, reliable, and effective cancer markers to assist in the diagnosis and treatment of NPC is of great significance.

It is well established that miRNAs are closely linked to oncogenesis and represent a potential tool for assisting cancer diagnosis and treatment.^29,31,32^ NPC cells could produce 2 types of miRNA. One type is produced by the host cell genome, namely, Homo sapiens miRNA (hsa-miRNA). The other is produced by the EBV genome, namely, EBV BART miRNA. For the former, miRNA profiling of hsa-miRNAs used to be studied in plasma and tumor tissues; however, absolutely no overlap was observed in these selected miRNA signatures in NPC.^33-35^ Hence, the prognostic value of hsa-miRNAs is markedly restricted. It is worth noting that EBV BART miRNAs are abundantly expressed in NPC and are relatively consistent regardless of microRNA sequencing of NPC biopsies or blood samples, indicating that EBV BART miRNAs represent promising biomarkers.^36^ Additionally, compared to the thousands of hsa-miRNAs, only 44 mature EBV BART miRNAs need to be detected. Therefore, utilizing EBV BART miRNAs in clinical practice is available and reliable.

To the best of our knowledge, this is the first study to characterize both pretreatment and post-treatment plasma BART8-3p in NPC patients. Our study found that circulating BART8-3p was higher in locally advanced NPC than in early NPC and almost undetectable in healthy donors, and BART8-3p was significantly related to NPC progression. Importantly, we confirmed that BART8-3p was a sensitive and specific biomarker for diagnosing NPC, with a predictive value of 92%. Thus, plasma BART8-3p has potential clinical value in diagnosing early-stage NPC. Gao et al^37^ reported that plasma BART8-3p was highest in recurrent NPC, and BART8-3p was not significantly expressed between noncancer subjects and NPC patients. The reasons for the discrepancies are unclear, but it is likely due to the differences in primer and test methods, which can also be seen in other studies that some BART miRNAs were differentially expressed.^38,39^ Additionally, we found that BART8-3p showed a weak correlation with the load of EBV DNA. More importantly, BART8-3p can be detected in plasma with undetectable EBV DNA in a few NPC patients. In contrast, few studies have shown that plasma BART miRNAs, such as BART7 and BART17-5p, are not associated with EBV DNA.^40,41^ Therefore, BART8-3p could be examined given that EBV DNA was not detectable in clinical practice.

NPC is a radiosensitive cancer, and RT is supposed to be the mainstay treatment. However, a significant portion of patients will develop locoregional recurrence or distant metastases after RT. Hirai et al^42^ reported that the copy numbers of serum BART2-5p, BART17-5p and BART18-5p showed no significant change after treatment, while post-treatment BART17-5p could be used as a biomarker in recurrent or residual NPC, indicating a poor prognosis. Zhang et al^21^ showed that circulating BART7 and BART13 were significantly decreased after treatment, while there was no change in circulating BART3. Interestingly, in line with miR-BART17-5p, BART7 and BART13, our study found that plasma BART8-3p was remarkably diminished after treatment. No significant decrease in BART8-3p or an increase in BART8-3p after a decrease to 0 copies/mL may be indicative of persistent tumor or metastasis. The prognostic analysis results indicated that high levels of pretreatment and post-treatment BART8-3p were all significantly associated with an increased likelihood of cancer recurrence and adverse prognosis. Thus, for patients with high levels of BART8-3p, especially those with high levels of post-treatment BART8-3p, extreme caution should be taken, and additional therapy might be needed.

The Intergroup 0099 study demonstrated that compared to RTRT alone, RT plus chemotherapy greatly improved NPC patient outcomes.^43^ Currently, concurrent chemoradiotherapy has been established as the standard treatment of LA-NPC.^44^ Our study found that LA-NPC with high levels of pretreatment or post-treatment BART8-3p was related to a poor prognosis, indicating that BART8-3p was a potential biomarker in LA-NPC. Currently, how many cycles of chemotherapy need to be given in LA-NPC remains a challenge, as LA-NPC has large heterogeneity. Our study found that for LA-NPC patients with high levels of pretreatment BART8-3p, more cycles of chemotherapy (≥6 cycles) tended to confer prolonged OS. Of note, more than 50% of LA-NPC patients with high post-treatment BART8-3p levels presented distant metastasis, suggesting the need for better interventions in this special group. Hence, plasma BART8-3p is a promising biomarker for predicting the prognosis and therapeutic benefit of enough thermotherapy in LA-NPC. More research is urgently needed to determine the utility of BART8-3p in clinical practice.

However, EBV miRNA/BART testing still faces a few challenges. First, due to the different miRNA sequencing methods or detection systems, the expression profiles, sensitivity, and specificity of BART miRNAs are somewhat different. In addition, a BART microRNA level that is higher than the suspected nonspecific reaction may indicate other EBV-infected diseases, such as lymphoma, gastric cancer, or chronic EBV infection.^45-47^ In addition, a small number of NPC patients are BART microRNA negative, possibly because a tiny minority of patients are EBV negative or because they have early-stage disease. Finally, due to the technical restrictions in EBV DNA detection prior to June 2016, the clinical utility of BART8-3p was not compared with that of EBV DNA in this article. Thus, further validation is needed in future prospective clinical trials. In addition, the value of plasma BART8-3p needs to be confirmed in multicenter and large-sample studies.

## Conclusions

We found that plasma BART8-3p can distinguish NPC patients from HCs with high sensitivity and specificity. Our results reveal that plasma BART8-3p is a meaningful biomarker for predicting the risk of recurrence and metastasis and has clinical value for the selection of suitable NPC therapy so that more NPC patients can benefit from the optimal therapy.

## Supplementary Material

oyac024_suppl_Supplementary_Table_S1

## Data Availability

The data underlying this article will be shared on reasonable request to the corresponding author.
