# Supplementary material for: Plasma Epstein-Barr Virus MicroRNA BART8-3p as a Diagnostic and Prognostic Biomarker in Nasopharyngeal Carcinoma
Source: Oncologist. 2022 Mar 5;27(4):e340–9. doi: 10.1093/oncolo/oyac024 (PMC8982379; doi:10.1093/oncolo/oyac024)
Supplement: oyac024_suppl_Supplementary_Table_S1 [file oyac024_suppl_supplementary_table_s1.pdf]

The official journal of the Society for Translational Oncology

Supplemental Table for:
Plasma Epstein–Barr virus microRNA BART8-3p as a diagnostic and prognostic biomarker in nasopharyngeal carcinoma
JianJi Pan et al.

Supplemental Table 1. miR-BART8-3p sequence and primer sequence for reverse transcription and quantitative real-time PCR

Category	Sequence
ebv miR-BART8-3p MIMAT0003418	5'- GUCACAAUCUAUGGGGUCGUAGA- 3'
BART8-3p TaqMan Probe	5'- CGCACTGGATACGACTCTACG - 3'
BART8-3p Forward primer	5'- ATCGTCACAATCTATGGGGT-3'
BART8-3p Reverse primer	5'- GCAGGGTCCGAGGTATTC-3'
BART8-3p Reverse transcription primer	GTCGTATCCAGTGCAGGGTCCGAGGTATTCGCACTGGATACGACTCTACG
